# Supplementary material for: Optimizing hierarchical tree dissection parameters using historic epidemiologic data as ‘ground truth’
Source: PLoS One. 2023 Feb 24;18(2):e0282154. doi: 10.1371/journal.pone.0282154 (PMC9955612; doi:10.1371/journal.pone.0282154)
Supplement: Attachment — Submitted filename: StringencyOptimization_ResponsetoReviewers.docx [file pone.0282154.s003.docx]

We appreciate the comments from the reviewers and we believe that we have addressed all of the comments listed below. Of interest, both reviewers suggested including additional performance metrics (e.g., specificity, precision, PPV) and this was a point of discussion between the authors before finalizing our initial submission. In our original submission, we ultimately decided to only include accuracy for the sake of brevity in the tables/text; however, it is clear that additional performance metrics would be useful, and we have now included sensitivity, specificity, PPV (i.e., precision), and NPV in our tables and results text in the revised manuscript.

A further note is that our group has published an update regarding species naming for human-infecting *Cyclospora* parasites while this present manuscript was in review. We have updated our *Cyclospora* species naming convention in the revised manuscript to state *Cyclospora* spp. or *Cyclospora* sp., rather than *Cyclospora cayetanensis*.

Please see our response to specific comments below.

Reviewer #1:

Comments on the manuscript are as follows:

1. - Why did the author omit the Simpson index results and did not add to a table of results like Table 2 for example?
   a. Simpson index results for all stringency values have been added to Table 2.
2. - The authors reported just the accuracy of the trees, why not add sensitivity specificity and precision as performance metrics as well to their results?
   a. Sensitivity, specificity, PPV, and NPV values have been added to Table 2 and the text results. Methods have been updated to mention calculation of these performance metrics.
3. - The quality of the figure is very high, however in my opinion, the results can be better presented if each tree A, B and C are presented in separate figures.
   a. This is a good point and we agree that splitting the figure into separate figures makes for easier interpretation. As such, Figure 1 A-C has been split into Fig 1, Fig 2, and Fig 3, respectively. Manuscript text and figure legends have been updated appropriately.

Reviewer #2:

1. In table 2, various performance metrics such as sensitivity, specificity, PPV, and NPV can be calculated and compared.
   a. Sensitivity, specificity, PPV, and NPV values have been added to Table 2 and the text results. Methods have been updated to mention calculation of these performance metrics.
2. Since choosing an appropriate distance statistic is fundamental, please explain why using Barratt’s heuristic definition of genetic distance is the best option.
   a. We have added text in the introduction section describing why Barratt’s heuristic was chosen to calculate genetic distances.
3. It seems that references 16 and 22 are the same. Please omit one of them.
   a. Thank you for the attention to detail. This issue has been resolved and the initial citation #22 has been removed
